# Supplementary material for: HIVprotI: an integrated web based platform for prediction and design of HIV proteins inhibitors
Source: J Cheminform. 2018 Mar 9;10:12. doi: 10.1186/s13321-018-0266-y (PMC5845081; doi:10.1186/s13321-018-0266-y)
Supplement: Supplementary file 2 — Additional file 2. Source code of HIVProtI web server. [file 13321_2018_266_MOESM2_ESM.zip › HIVprotI_Source-code/hivprotI/weka-3-6-6/doc/weka/gui/beans/BeanVisual.html]

BeanVisual


---


|  |  |  |
| --- | --- | --- |
| **Overview** \| **Package** \| **Class** \| **Tree** \| **Deprecated** \| **Index** \| **Help** \| **Weka's home** | |  |
| **PREV CLASS**   **NEXT CLASS** | **FRAMES**    **NO FRAMES**     **All Classes** |  |
| SUMMARY: NESTED \| FIELD \| CONSTR \| METHOD | DETAIL: FIELD \| CONSTR \| METHOD |  |


---


## weka.gui.beans Class BeanVisual

```
java.lang.Object
  java.awt.Component
      java.awt.Container
          javax.swing.JComponent
              javax.swing.JPanel
                  weka.gui.beans.BeanVisual
```

**All Implemented Interfaces:**: java.awt.image.ImageObserver, java.awt.MenuContainer, java.io.Serializable, javax.accessibility.Accessible

---

``` public class BeanVisual extends javax.swing.JPanel ```

BeanVisual encapsulates icons and label for a given bean. Has methods
to load icons, set label text and toggle between static and animated
versions of a bean's icon.

**Since:**
:   1.0

**Version:**
:   $Revision: 1.10 $

**Author:**
:   Mark Hall

**See Also:**: `JPanel`, `Serializable`, Serialized Form

---

**Nested Class Summary**

**Nested classes/interfaces inherited from class javax.swing.JComponent**

`javax.swing.JComponent.AccessibleJComponent`

**Nested classes/interfaces inherited from class java.awt.Component**

`java.awt.Component.BaselineResizeBehavior`


| **Field Summary** | |
| --- | --- |
| `static int` | `EAST_CONNECTOR` |
| `static java.lang.String` | `ICON_PATH` |
| `static int` | `NORTH_CONNECTOR` |
| `static int` | `SOUTH_CONNECTOR` |
| `static int` | `WEST_CONNECTOR` |

**Fields inherited from class javax.swing.JComponent**

`TOOL_TIP_TEXT_KEY, UNDEFINED_CONDITION, WHEN_ANCESTOR_OF_FOCUSED_COMPONENT, WHEN_FOCUSED, WHEN_IN_FOCUSED_WINDOW`

**Fields inherited from class java.awt.Component**

`BOTTOM_ALIGNMENT, CENTER_ALIGNMENT, LEFT_ALIGNMENT, RIGHT_ALIGNMENT, TOP_ALIGNMENT`

**Fields inherited from interface java.awt.image.ImageObserver**

`ABORT, ALLBITS, ERROR, FRAMEBITS, HEIGHT, PROPERTIES, SOMEBITS, WIDTH`


**Constructor Summary**

`BeanVisual(java.lang.String visualName,
java.lang.String iconPath,
java.lang.String animatedIconPath)`
  
          Constructor


| **Method Summary** | |
| --- | --- |
| `void` | `addPropertyChangeListener(java.beans.PropertyChangeListener pcl)`             Add a listener for property change events |
| `javax.swing.ImageIcon` | `getAnimatedIcon()`             Returns the animated icon |
| `java.lang.String` | `getAnimatedIconPath()`             returns the path for the animated icon |
| `java.awt.Point` | `getClosestConnectorPoint(java.awt.Point pt)`             Returns the coordinates of the closest "connector" point to the supplied point. |
| `java.awt.Point` | `getConnectorPoint(int compassPoint)`             Returns the coordinates of the connector point given a compass point |
| `java.lang.String` | `getIconPath()`             returns the path for the icon |
| `javax.swing.ImageIcon` | `getStaticIcon()`             Returns the static icon |
| `java.lang.String` | `getText()`             Get the visual's label |
| `boolean` | `loadIcons(java.lang.String iconPath, java.lang.String animatedIconPath)`             Loads static and animated versions of a beans icons. |
| `void` | `paintComponent(java.awt.Graphics gx)` |
| `void` | `removePropertyChangeListener(java.beans.PropertyChangeListener pcl)`             Remove a property change listener |
| `void` | `scale(int factor)`             Reduce this BeanVisual's icon size by the given factor |
| `void` | `setAnimated()`             Set the animated version of the icon |
| `void` | `setDisplayConnectors(boolean dc)`             Turn on/off the connector points |
| `void` | `setDisplayConnectors(boolean dc, java.awt.Color c)`             Turn on/off the connector points |
| `void` | `setStatic()`             Set the static version of the icon |
| `void` | `setText(java.lang.String text)`             Set the label for the visual. |

**Methods inherited from class javax.swing.JPanel**

`getAccessibleContext, getUI, getUIClassID, setUI, updateUI`

**Methods inherited from class javax.swing.JComponent**

`addAncestorListener, addNotify, addVetoableChangeListener, computeVisibleRect, contains, createToolTip, disable, enable, firePropertyChange, firePropertyChange, firePropertyChange, getActionForKeyStroke, getActionMap, getAlignmentX, getAlignmentY, getAncestorListeners, getAutoscrolls, getBaseline, getBaselineResizeBehavior, getBorder, getBounds, getClientProperty, getComponentPopupMenu, getConditionForKeyStroke, getDebugGraphicsOptions, getDefaultLocale, getFontMetrics, getGraphics, getHeight, getInheritsPopupMenu, getInputMap, getInputMap, getInputVerifier, getInsets, getInsets, getListeners, getLocation, getMaximumSize, getMinimumSize, getNextFocusableComponent, getPopupLocation, getPreferredSize, getRegisteredKeyStrokes, getRootPane, getSize, getToolTipLocation, getToolTipText, getToolTipText, getTopLevelAncestor, getTransferHandler, getVerifyInputWhenFocusTarget, getVetoableChangeListeners, getVisibleRect, getWidth, getX, getY, grabFocus, isDoubleBuffered, isLightweightComponent, isManagingFocus, isOpaque, isOptimizedDrawingEnabled, isPaintingForPrint, isPaintingTile, isRequestFocusEnabled, isValidateRoot, paint, paintImmediately, paintImmediately, print, printAll, putClientProperty, registerKeyboardAction, registerKeyboardAction, removeAncestorListener, removeNotify, removeVetoableChangeListener, repaint, repaint, requestDefaultFocus, requestFocus, requestFocus, requestFocusInWindow, resetKeyboardActions, reshape, revalidate, scrollRectToVisible, setActionMap, setAlignmentX, setAlignmentY, setAutoscrolls, setBackground, setBorder, setComponentPopupMenu, setDebugGraphicsOptions, setDefaultLocale, setDoubleBuffered, setEnabled, setFocusTraversalKeys, setFont, setForeground, setInheritsPopupMenu, setInputMap, setInputVerifier, setMaximumSize, setMinimumSize, setNextFocusableComponent, setOpaque, setPreferredSize, setRequestFocusEnabled, setToolTipText, setTransferHandler, setVerifyInputWhenFocusTarget, setVisible, unregisterKeyboardAction, update`

**Methods inherited from class java.awt.Container**

`add, add, add, add, add, addContainerListener, addPropertyChangeListener, applyComponentOrientation, areFocusTraversalKeysSet, countComponents, deliverEvent, doLayout, findComponentAt, findComponentAt, getComponent, getComponentAt, getComponentAt, getComponentCount, getComponents, getComponentZOrder, getContainerListeners, getFocusTraversalKeys, getFocusTraversalPolicy, getLayout, getMousePosition, insets, invalidate, isAncestorOf, isFocusCycleRoot, isFocusCycleRoot, isFocusTraversalPolicyProvider, isFocusTraversalPolicySet, layout, list, list, locate, minimumSize, paintComponents, preferredSize, printComponents, remove, remove, removeAll, removeContainerListener, setComponentZOrder, setFocusCycleRoot, setFocusTraversalPolicy, setFocusTraversalPolicyProvider, setLayout, transferFocusBackward, transferFocusDownCycle, validate`

**Methods inherited from class java.awt.Component**

`action, add, addComponentListener, addFocusListener, addHierarchyBoundsListener, addHierarchyListener, addInputMethodListener, addKeyListener, addMouseListener, addMouseMotionListener, addMouseWheelListener, bounds, checkImage, checkImage, contains, createImage, createImage, createVolatileImage, createVolatileImage, dispatchEvent, enable, enableInputMethods, firePropertyChange, firePropertyChange, firePropertyChange, firePropertyChange, firePropertyChange, getBackground, getBounds, getColorModel, getComponentListeners, getComponentOrientation, getCursor, getDropTarget, getFocusCycleRootAncestor, getFocusListeners, getFocusTraversalKeysEnabled, getFont, getForeground, getGraphicsConfiguration, getHierarchyBoundsListeners, getHierarchyListeners, getIgnoreRepaint, getInputContext, getInputMethodListeners, getInputMethodRequests, getKeyListeners, getLocale, getLocation, getLocationOnScreen, getMouseListeners, getMouseMotionListeners, getMousePosition, getMouseWheelListeners, getName, getParent, getPeer, getPropertyChangeListeners, getPropertyChangeListeners, getSize, getToolkit, getTreeLock, gotFocus, handleEvent, hasFocus, hide, imageUpdate, inside, isBackgroundSet, isCursorSet, isDisplayable, isEnabled, isFocusable, isFocusOwner, isFocusTraversable, isFontSet, isForegroundSet, isLightweight, isMaximumSizeSet, isMinimumSizeSet, isPreferredSizeSet, isShowing, isValid, isVisible, keyDown, keyUp, list, list, list, location, lostFocus, mouseDown, mouseDrag, mouseEnter, mouseExit, mouseMove, mouseUp, move, nextFocus, paintAll, postEvent, prepareImage, prepareImage, remove, removeComponentListener, removeFocusListener, removeHierarchyBoundsListener, removeHierarchyListener, removeInputMethodListener, removeKeyListener, removeMouseListener, removeMouseMotionListener, removeMouseWheelListener, removePropertyChangeListener, repaint, repaint, repaint, resize, resize, setBounds, setBounds, setComponentOrientation, setCursor, setDropTarget, setFocusable, setFocusTraversalKeysEnabled, setIgnoreRepaint, setLocale, setLocation, setLocation, setName, setSize, setSize, show, show, size, toString, transferFocus, transferFocusUpCycle`

**Methods inherited from class java.lang.Object**

`equals, getClass, hashCode, notify, notifyAll, wait, wait, wait`

**Field Detail**

### ICON\_PATH

```
public static final java.lang.String ICON_PATH
```

**See Also:**: Constant Field Values

---


### NORTH\_CONNECTOR

```
public static final int NORTH_CONNECTOR
```

**See Also:**: Constant Field Values

---


### SOUTH\_CONNECTOR

```
public static final int SOUTH_CONNECTOR
```

**See Also:**: Constant Field Values

---


### EAST\_CONNECTOR

```
public static final int EAST_CONNECTOR
```

**See Also:**: Constant Field Values

---


### WEST\_CONNECTOR

```
public static final int WEST_CONNECTOR
```

**See Also:**: Constant Field Values


**Constructor Detail**

### BeanVisual

```
public BeanVisual(java.lang.String visualName,
                  java.lang.String iconPath,
                  java.lang.String animatedIconPath)
```

:   Constructor

    **Parameters:**: `visualName` - name for the bean: `iconPath` - path to the icon file: `animatedIconPath` - path to the animated icon file


**Method Detail**

### scale

```
public void scale(int factor)
```

:   Reduce this BeanVisual's icon size by the given factor

    :   **Parameters:**: `factor` - the factor by which to reduce the icon size by

---


### loadIcons

```
public boolean loadIcons(java.lang.String iconPath,
                         java.lang.String animatedIconPath)
```

:   Loads static and animated versions of a beans icons. These are
    assumed to be defined in the system resource location (i.e. in the
    CLASSPATH). If the named icons do not exist, no changes to the
    visual appearance is made. Since default icons for generic
    types of beans (eg. DataSource, Classifier etc)
    are assumed to exist, it allows developers to add custom icons for
    for specific instantiations of these beans
    (eg. J48, DiscretizeFilter etc) at their leisure.

    :   **Parameters:**: `iconPath` - path to: `animatedIconPath` - a `String` value

---


### setText

```
public void setText(java.lang.String text)
```

:   Set the label for the visual. Informs any property change listeners

    :   **Parameters:**: `text` - the label

---


### getText

```
public java.lang.String getText()
```

:   Get the visual's label

    :   **Returns:**: a `String` value

---


### setStatic

```
public void setStatic()
```

:   Set the static version of the icon

---


### setAnimated

```
public void setAnimated()
```

:   Set the animated version of the icon

---


### getClosestConnectorPoint

```
public java.awt.Point getClosestConnectorPoint(java.awt.Point pt)
```

:   Returns the coordinates of the closest "connector" point to the
    supplied point. Coordinates are in the parent containers coordinate
    space.

    :   **Parameters:**: `pt` - the reference point **Returns:**: the closest connector point

---


### getConnectorPoint

```
public java.awt.Point getConnectorPoint(int compassPoint)
```

:   Returns the coordinates of the connector point given a compass point

    :   **Parameters:**: `compassPoint` - a compass point **Returns:**: a `Point` value

---


### getStaticIcon

```
public javax.swing.ImageIcon getStaticIcon()
```

:   Returns the static icon

    :   **Returns:**: an `ImageIcon` value

---


### getAnimatedIcon

```
public javax.swing.ImageIcon getAnimatedIcon()
```

:   Returns the animated icon

    :   **Returns:**: an `ImageIcon` value

---


### getIconPath

```
public java.lang.String getIconPath()
```

:   returns the path for the icon

    :   **Returns:**: the path for the icon

---


### getAnimatedIconPath

```
public java.lang.String getAnimatedIconPath()
```

:   returns the path for the animated icon

    :   **Returns:**: the path for the animated icon

---


### setDisplayConnectors

```
public void setDisplayConnectors(boolean dc)
```

:   Turn on/off the connector points

    :   **Parameters:**: `dc` - a `boolean` value

---


### setDisplayConnectors

```
public void setDisplayConnectors(boolean dc,
                                 java.awt.Color c)
```

:   Turn on/off the connector points

    :   **Parameters:**: `dc` - a `boolean` value: `c` - the Color to use

---


### addPropertyChangeListener

```
public void addPropertyChangeListener(java.beans.PropertyChangeListener pcl)
```

:   Add a listener for property change events

    :   **Overrides:**: `addPropertyChangeListener` in class `java.awt.Container`
    :   **Parameters:**: `pcl` - a `PropertyChangeListener` value

---


### removePropertyChangeListener

```
public void removePropertyChangeListener(java.beans.PropertyChangeListener pcl)
```

:   Remove a property change listener

    :   **Overrides:**: `removePropertyChangeListener` in class `java.awt.Component`
    :   **Parameters:**: `pcl` - a `PropertyChangeListener` value

---


### paintComponent

```
public void paintComponent(java.awt.Graphics gx)
```

:   **Overrides:**: `paintComponent` in class `javax.swing.JComponent`


---


|  |  |  |
| --- | --- | --- |
| **Overview** \| **Package** \| **Class** \| **Tree** \| **Deprecated** \| **Index** \| **Help** \| **Weka's home** | |  |
| **PREV CLASS**   **NEXT CLASS** | **FRAMES**    **NO FRAMES**     **All Classes** |  |
| SUMMARY: NESTED \| FIELD \| CONSTR \| METHOD | DETAIL: FIELD \| CONSTR \| METHOD |  |


---
